# Approaches to the Management of Metastatic Adenoid Cystic Carcinoma

**DOI:** 10.3390/cancers14225698

**Published:** 2022-11-20

**Authors:** Rex H. Lee, Katherine C. Wai, Jason W. Chan, Patrick K. Ha, Hyunseok Kang

**Affiliations:** 1 Department of Otolaryngology-Head and Neck Surgery, University of California San Francisco, San Francisco, CA 94143, USA; 2 Department of Otolaryngology-Head and Neck Surgery, Stanford University, Palo Alto, CA 94304, USA; 3 Department of Radiation Oncology, University of California San Francisco, San Francisco, CA 94143, USA; 4 Department of Medicine, University of California San Francisco, San Francisco, CA 94143, USA

**Keywords:** adenoid cystic carcinoma (ACC), salivary gland cancer, metastasis, systemic therapy, chemotherapy, tyrosine kinase inhibitor (TKI), immunotherapy, metastasectomy, radiation, SBRT

## Abstract

**Simple Summary:**

Patients with adenoid cystic carcinoma (ACC) often experience late distant metastasis years after definitive therapy, most commonly to the lungs. Currently, there is little consensus on the optimal treatment regimens for metastatic ACC, which typically confer modest clinical benefit. Here, we outline management approaches for metastatic ACC in the context of pertinent ACC biology. We summarize the most commonly utilized systemic treatment regimens, review methods of local control for oligometastatic lung disease, and highlight emerging molecular targets with promise for advancing ACC management in the future.

**Abstract:**

High rates of recurrence and distant metastasis are a foremost challenge in the management of adenoid cystic carcinoma (ACC), occurring in approximately 40% of all ACC patients. Despite the morbidity and mortality resulting from recurrent/metastatic (R/M) disease, there are no FDA-approved systemic agents for these patients. In this review, we summarize pertinent ACC pathophysiology and its implications for different systemic treatment regimens in R/M ACC. We review the evidence for the most widely used systemic agents — cytotoxic chemotherapy and tyrosine kinase inhibitors (TKIs) targeting VEGFR — in addition to immune checkpoint inhibitors and non-TKI biologic agents. Exciting emerging targets for R/M ACC, including inhibitors of Notch signaling, stemness, PRMT5, and Axl, are also discussed. Lastly, we review local therapies for small-volume lung disease in patients with oligometastatic ACC, specifically pulmonary metastasectomy and stereotactic body radiation therapy (SBRT). Future development of targeted molecular agents which exploit the underlying biology of this disease may yield novel therapeutic options to improve clinical outcomes in patients with R/M ACC.

## 1. Introduction

Adenoid cystic carcinoma (ACC) is a rare malignancy that accounts for just 1% of head and neck cancers, but represents approximately 25% of all salivary gland malignancies [1,2,3]. More than half of ACC arises from the minor salivary glands, with the hard palate being the most commonly affected intraoral site [1]. Within the head and neck, ACC can also affect the major salivary glands, paranasal sinuses, larynx, and trachea [4]. Most cases are diagnosed in the fifth or sixth decade of life, with a slight predilection for female sex [5].

Primary ACC is typically indolent and slow-growing, and can be successfully treated with surgical resection similarly to other salivary gland tumors. However, ACC often displays unpredictable and aggressive long-term behavior. In contrast to many head and neck cancers (HNC), lymphatic spread resulting in clinical nodal disease is rare in ACC [6]. However, perineural invasion (PNI) is strikingly common even in early tumors [7,8]. The high rate of PNI, coupled with perineural tumor spread and a propensity for hematogenous dissemination, make distant metastasis the most common pattern of treatment failure in ACC, often occurring in the absence of locoregional recurrence [9]. An estimated 30–40% of ACC patients will develop distant metastasis within 10–15 years following curative-intent treatment, most commonly to the lungs [9,10].

The high rate of recurrence and distant metastasis in ACC necessitates effective systemic and/or local therapies capable of durable disease control. Currently, there are no FDA-approved agents for recurrent/metastatic (R/M) ACC, which has resulted in highly variable treatment regimens across patients and institutions. The clinical and biological heterogeneity of ACC, coupled with an incomplete understanding of its molecular pathogenesis, have limited the development of successful targeted agents to date. The aim of this review is to summarize our current knowledge of the biology and molecular landscape of ACC, and to discuss current and emerging therapies for R/M ACC.

## 2. Biology of ACC

### 2.1. Cellular Origin and Histopathology

**Figure 1:**
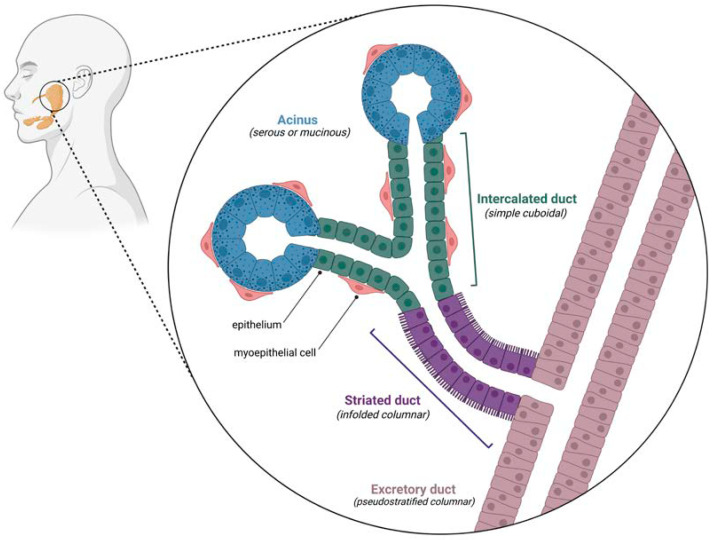
Schematic illustrating the spatial arrangement of the salivary ductal system.

Salivary glands have complex spatial arrangements of multiple diverse cell types. In particular, the salivary ductal system contains well-defined subdivisions which are relevant for understanding the cellular origin of ACC [11]. The smallest diameter ducts are the intercalated ducts, which are located directly adjacent to acini (the functional units of saliva production). Intercalated ducts empty into the striated ducts, which themselves lead to the larger interlobular excretory ducts that carry saliva to the oral cavity [12]. The arrangement of these ductal segments is depicted in Figure 1.

The epithelium of each ductal segment is phenotypically distinct, from simple cuboidal cells in the intercalated ducts, to extensively infolded columnar cells in the striated ducts, and a transition from pseudostratified columnar to stratified squamous epithelium as the excretory ducts approach the intraoral opening [12]. ACC arises from the intercalated ducts, which contain an outer layer of contractile myoepithelial cells in addition to the luminal cuboidal epithelial layer [13]. The presence of both epithelial and myoepithelial components is a unique characteristic of most ACCs, possibly due to the bidirectional differentiation of salivary gland stem cells (which largely reside in the intercalated ducts) [14]. Based on the relative proportion of these two cell types, ACC is classified as principally of luminal epithelial differentiation or myoepithelial differentiation, the latter of which is far more common [15,16].

Histologically, ACC is categorized into three major subtypes: cribriform, tubular, and solid [17]. The cribriform subtype is the most common, with histology characterized by variably sized nests of neoplastic cells interspersed with cystic spaces in a “swiss-cheese” pattern [15]. The predominantly myoepithelial differentiation of most cribriform and tubular ACC is hypothesized to contribute to the indolent and more restrained behavior of these subtypes. In contrast, the loss of myoepithelial cells is a hallmark of the solid subtype of ACC, which demonstrates a significantly more aggressive clinical course and higher propensity for metastatic spread [18,19].

### 2.2. Genomic Landscape

Compared to other HNC types such as squamous cell carcinoma (HNSCC), the genetic underpinnings of ACC remain poorly understood. The most prevalent mutations in HNSCC (including *TP53* and *CDKN2A* in HPV- HNSCC and *PIK3CA* in HPV+ HNSCC) are found in an exceedingly low proportion of ACCs [20,21]. A rarity of mutations in otherwise common oncogenes and tumor suppressors have led to the hypothesis that ACC oncogenesis may rely more heavily on transcriptional alterations and changes in chromatin structure than other HNC types [22]. Here, we briefly describe the three most common mutational patterns reported in primary and R/M ACC: those affecting *MYB/MYBL1*, members of the Notch signaling pathway, and genes involved in chromatin regulation.

#### 2.2.1. MYB/MYBL1

There has been great interest in the myeloblastosis oncogene (*MYB*) in ACC since 2009, when Persson et al. reported a characteristic translocation between *MYB* and the nuclear factor 1B gene (*NFIB*) in these tumors [23]. This t(6;9)(q22–23;p23–24) translocation is the only recurrent structural aberration reliably reported in primary ACC, and is present in approximately 60% of cases [24]. In contrast, only 22% of R/M ACC demonstrate *MYB* or *MYBL1* rearrangements. It is important to note that the MYB protein is overexpressed in upwards of 80% of all ACC, suggesting a central role for MYB in ACC’s pathogenesis even in the absence of a *MYB-NFIB* fusion [25]. Contrary to initial studies, the cumulative evidence suggests that neither translocation nor overexpression of MYB carries meaningful prognostic significance in ACC [26].

#### 2.2.2. Notch Signaling

Binding of Notch ligands with Notch receptors triggers a cascade of consecutive receptor cleavages, including a gamma-secretase release of the Notch intracellular domain (NICD), which regulates downstream transcription [27]. In general, genetic alterations of the Notch pathway are present in about 13% of primary ACC, most commonly mutations in *NOTCH1* (8% of primary ACC) [24]. Less commonly, mutations in *NOTCH2-4* are found, as well as genes encoding key downstream proteins in the signaling pathway such as *SPEN* (an NICD corepressor) and *FBXW7* (a component of the ubiquitin-proteasome system), among others [21,24]. Compared to primary ACC, mutations in genes of the Notch pathway are far more common in R/M ACC, occurring in approximately 40% of all cases [28]. Strikingly, 26% of R/M ACC display mutations in *NOTCH1* specifically [28]. Recent work has associated activating *NOTCH1* mutations with poor prognosis and increased rates of liver and bone metastasis in ACC [29]. Upregulation of Notch signaling has been shown to suppress myoepithelial differentiation in ACC, a characteristic of the solid subtype, and may contribute to the unrestrained clinical behavior of these cases [30]. It is worth mentioning that in R/M ACC, mutations in *NOTCH1* and *TERT* promoters are largely mutually exclusive, with *TERT*-mutated ACC also generally lacking *MYB* rearrangements. These observations suggest a distinct *TERT*-driven subgroup with potentially separate oncogenic pathways and treatment susceptibilities [28].

#### 2.2.3. Chromatin State Regulators

Mutations in chromatin remodeling genes are present in 35% of primary ACC [24]. Among the most conserved alterations are mutations in the histone demethylase *KDM6A* (7%), the histone acetyltransferase *CREBBP* (7%), and *SMARCA2* (5%), which encodes the central ATPase of the SWI/SNF remodeling complex [24,31,32]. As with Notch signaling, alterations in chromatin remodeling genes are significantly more common in R/M ACC than primary tumors, including *KDM6A* mutations in 15% and *CREBBP* mutations in 11%, as well as other members of the SWI/SNF complex (i.e., the *ARID* family) [28,32]. In contrast to the mutual exclusivity of *NOTCH1* and *TERT* promoter mutations in R/M ACC, there is a conspicuous co-occurrence of mutations in *NOTCH1* and chromatin remodeling genes in these patients (particularly *KDM6A*, *CREBBP*, and *ARID1A*) [28]. This suggests that the role of Notch signaling in ACC progression may be promoted by upstream epigenetic reprogramming via chromatin state regulators [22].

### 2.3. Immune Microenvironment

ACC is generally considered an immunologically “cold” tumor, with a low density of dendritic cells and CD8+ tumor-infiltrating lymphocytes (TILs) [33]. The unremarkable immune infiltrate seen in most ACCs is likely related to its low tumor mutational burden (TMB), which results in an insufficient neoantigen load for triggering a robust adaptive immune response [34]. One study comparing the immune landscape between different types of salivary gland cancers found that ACC had the least conducive immune profile to immunotherapy, with a low TMB (0.3 mutations/megabase), loss of HLA class I, and a T-cell exclusion phenotype stemming from increased myeloid-derived suppressor cells and inhibitory M2 macrophages [35].

The most commonly used immune checkpoint inhibitors (ICIs) in HNC target signaling between the programmed death-1 receptor (PD-1) and programmed death ligands (PD-Ls). Pembrolizumab and nivolumab, monoclonal antibodies which inhibit PD-1, enhance tumor recognition by CD8+ TILs and stimulate cancer cell destruction [36]. The combined positive score (CPS) is a standard method for identifying HNC patients who are most likely to benefit from anti-PD-1 ICIs, and is determined through immunohistochemical analysis of the proportion of PD-L1-positive cells (including cancer cells, lymphocytes, and macrophages) in relation to total tumor cells in a patient’s sample [37]. Compared to HNSCC, PD-L1 expression is exceedingly low in the vast majority of ACCs. Three separate studies of primary and R/M ACC reported that none of the samples analyzed were significantly PD-L1-positive by immunohistochemistry (*n* = 36, 21, and 24); tumors with CPS ≥ 1 are thus a significant minority of ACC patients [33,38,39].

In contrast to consistently low PD-L1 positivity, robust PD-L2 expression has been reported in a high proportion (60–86%) of both primary and R/M ACC [38,40]. For this reason, PD-L2 has been hypothesized as a major driver of immune evasion in ACC [33]. Compared to the ubiquitous use of the PD-L1 CPS for ICI response prediction, the significance of PD-L2 positivity in predicting benefit from PD-1 inhibitors has been underexplored. Importantly, PD-L2 status was shown to predict clinical response to pembrolizumab independently of PD-L1 expression in 172 patients with HNSCC, though evaluation of PD-L2 is not regularly employed when considering ICI candidacy [41]. These observations lend further support for the use of PD-1 targeted therapies (pembrolizumab, nivolumab, and cemiplimab), as compared to PD-L1 inhibitors (atezolizumab, avelumab, and durvalumab), for the treatment of R/M ACC.

## 3. Adjuvant Chemoradiation

The preferred primary treatment modality for salivary malignancies, including ACC, remains curative-intent surgical resection [42]. At present, radiation therapy (RT) and chemotherapy play a minor role in adjuvant settings, often based on early, preliminary results. This is in contrast to aggressive, locally advanced HNSCC, in which the benefit of adjuvant concurrent chemoradiation is well established [43,44]. Most studies that do address the use of adjuvant RT plus systemic therapy include a heterogeneous group of salivary gland histologies and do not focus solely on ACC. Further, there is no standard of care for the dosing or type of chemotherapeutic agent administered, though most regimens include platinum-based therapy.

In a retrospective study by Mifsud et al., the authors found that in all subtypes of salivary gland cancer, there was no difference in overall survival (OS) or progression-free survival (PFS) among patients treated with adjuvant RT compared to adjuvant chemoradiation [45,46]. A separate study, again including all subtypes of salivary gland cancer, found that patients treated with adjuvant chemoradiation were more likely to have aggressive pathologic features including positive margins, nodal involvement, and lymphovascular invasion, compared to patients chosen for adjuvant RT alone [47]. In the chemoradiation group, the authors demonstrated good local control, with only 1/22 developing a local failure. Notably, this single locoregional failure was a patient with ACC. Regarding studies focused on ACC alone, a single-institution, retrospective study found no difference in OS among patients treated with surgery alone (*n* = 4), surgery + adjuvant RT (*n* = 21) and surgery + chemoradiation (*n* = 4) [48]. However, given the small sample size, these results were not adjusted for other important variables. A larger study utilizing propensity-score matching showed that in 91 patients with ACC, adjuvant concurrent chemoradiation compared to RT alone was associated with improved 5- and 8-year locoregional control, though there was no difference in disease-free survival (DFS) or OS [49].

Overall, there remains no consensus regarding the addition of adjuvant chemotherapy for high-risk ACC or any salivary gland cancer. RTOG-1008 is an active clinical trial (NCT01220583) aimed to fill this gap in our knowledge. In this trial, patients with high-risk salivary gland malignancies are randomized after surgical resection to either adjuvant RT or adjuvant chemoradiation with cisplatin. The primary outcome is PFS at 2 years. This will be the first prospective study to address this question about adjuvant treatment.

## 4. Systemic Agents for Recurrent/Metastatic ACC

**Figure 2:**
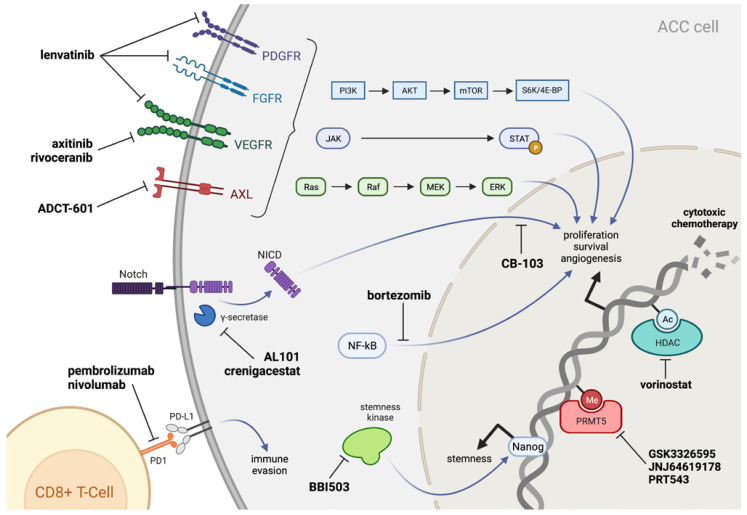
Summary of the mechanisms of systemic agents discussed in this review.

It is important to note that not all R/M ACC patients require active systemic treatment. If the disease is amenable to local treatment measures such as surgery or RT, those can be attempted to delay the start of systemic therapy. Salvage surgery for locoregional recurrence in ACC has been associated with significantly increased long-term survival in a study with over 25 years of follow-up [50], suggesting that locoregional control is still worthwhile in patients with limited metastasis, as it is difficult to predict how progressive the distant disease will be. For select patients who have indolent disease without symptoms, active surveillance can be offered as well. Although there are no FDA-approved systemic therapy options available for ACC, most clinicians use multi-kinase inhibitors targeting vascular endothelial growth factor receptor (VEGFR) pathways, as agents including lenvatinib and axitinib have demonstrated modest clinical benefit in these patients. Here, we review the evidence base for regimens of previously reported systemic agents in R/M ACC. The sites and mechanisms of action for the current and emerging systemic therapies reviewed in this article are depicted in Figure 2.

### 4.1. Cytotoxic Chemotherapy

Chemotherapy is generally reserved for palliative treatment in patients with symptomatic R/M ACC. Although several prospective and retrospective studies have been performed in this setting, there is no consensus for the optimal treatment regimen [51]. Overall objective response rates typically do not exceed ~20%. Furthermore, experimental studies with these therapies are often limited by sample size.

Combination therapy with cyclophosphamide-doxorubicin-cisplatin (CAP) is the most well-studied combination therapy, with an overall objective response rate up to 33% [52,53]. Described in the 1980s, a series of 13 advanced salivary gland malignancies showed that 3/9 (33%) of patients with ACC exhibited a response to CAP therapy [54]. A similarly small case series published in 2020 demonstrated that among R/M ACC patients treated with CAP, 2/14 achieved partial response (PR) and 10/14 showed stable disease (SD), with a median OS of 23.4 months [55]. Given the overall small number of patients in each study, treatment of R/M ACC with CAP requires larger studies to determine its role in this disease.

Multiple additional platinum combination therapies have been tested in ACC. A phase 2 clinical study investigated the efficacy of gemcitabine and cisplatin (or carboplatin among cisplatin-ineligible patients) in all R/M salivary gland malignancies, not exclusive to ACC [56]. Only 2/10 patients with ACC showed an objective response, though notably a separate study of gemcitabine alone in R/M ACC showed no objective responses [57]. Similarly, a study of carboplatin and paclitaxel across multiple histologic subtypes of R/M salivary gland cancer reported an objective response in 39% of all patients, but only 9% of those with ACC [58]. Outside of CAP, the regimen with the most available evidence is cisplatin and vinorelbine, which has yielded more encouraging results, with three studies reporting objective responses in 7/34 (21%), 6/22 (27%), and 6/19 (32%) of R/M ACC patients [59,60,61].

Eribulin, a non-taxane microtubule inhibitor currently approved for the treatment of metastatic breast cancer and metastatic liposarcoma, has also been of interest in ACC. Between 2012–2015, 29 patients with R/M salivary gland malignancies were enrolled in a phase 2 clinical trial with eribulin [62]. Eleven patients with R/M ACC were included; 1/11 had a complete response (CR) and 1/11 had a PR. Notably, 79% of patients included in the study had SD. More recently, a phase 1 study of the liposomal formulation of eribulin (E7389-LF) was tested in Japan in patients with advanced, nonresectable, or recurrent solid tumors [63]. In terms of efficacy with ACC, 2/12 achieved PR and 9/12 achieved SD, with a median PFS of 16.6 months. Although not designed to compare to other tumor types, the average PFS among the other malignancies was <4 months.

In summary, though cytotoxic chemotherapy regimens (especially platinum-based) have historically been employed for R/M ACC with marginal benefit, the use of these agents is based on studies with exceedingly small sample sizes, and thus the overall role of chemotherapy requires additional investigation.

### 4.2. Tyrosine Kinase Inhibitors

Tyrosine kinase receptors include a broad range of receptors instrumental for cell regulation and survival. One of these receptor families, the vascular endothelial growth factor receptors (VEGFRs), plays a key role in angiogenesis and lymphangiogenesis in cancer, and is an active area of research. Of the three main VEGF receptors (VEGFR-1, VEGFR-2, and VEGFR-3), the majority of VEGF-mediated angiogenic signaling occurs through VEGFR-2 [64]. In ACC, the expression of VEGF has been correlated with worse survival, making VEGFRs a possible therapeutic target [65,66]. Multiple preliminary clinical studies have been undertaken in patients with R/M ACC with varying degrees of success. We will present those with the most promising results below.

Axitinib is a multikinase inhibitor that is thought to be primarily an inhibitor of VEGFR-1, -2, and -3. A phase 2 clinical trial for patients with incurable ACC found that patients treated with axitinib had PR in 9% and SD >6 months in 75% [67]. Recently, a randomized phase 2 trial of 60 patients with R/M ACC demonstrated an improved 6-month PFS in patients who received axitinib compared to the observation group (10.8 vs. 2.8 months, respectively) [68].

Lenvatinib is another multikinase inhibitor with activity against all three VEGFRs, as well as fibroblast growth factor receptor (FGFR), platelet-derived growth factor receptor (PDGFR), KIT and RET. A phase 2 clinical trial in 2019 enrolled patients with R/M ACC with evidence of either radiographic or clinical disease progression at the time of enrollment [69]. Of 32 patients, 5/32 (16%) had PR and 24/32 (75%) had SD. Similar results were demonstrated in a similar study, with PR in 3/26 (11%) patients [70].

Another drug being tested is rivoceranib (also known as apatinib), a small molecule inhibitor of VEGFR-2. A prospective phase 2 clinical trial of this agent was carried out in China for R/M ACC. Among 65 analyzable patients, the objective response rate was 46% and the disease control rate was 98%, with a median 1-year OS of 92% [71]. A similar phase 2 study was undertaken in the United States and South Korea which did not demonstrate the same promising results, reporting an objective response rate of 13–19%, but the study only included patients with documented radiographic progression at the time of enrollment and yet demonstrated robust activity in R/M ACC [72]. Additional studies are needed to confirm the utility of rivoceranib in the treatment of ACC.

Overall, early phase 1/2 clinical trials of multi-kinase inhibitors targeting VEGFR have demonstrated modest but encouraging clinical benefits in R/M ACC. These targeted treatments may be considered over cytotoxic chemotherapy, especially given the comparatively tolerable toxicity profile.

### 4.3. Immunotherapy

Immune checkpoint inhibitor (ICI) therapy has shown promise in advanced head and neck cancers, though the overall clinical benefit in ACC is less clear with generally poor response rates thus far. Prior work suggests that the overall low tumor mutational burden and low PD-L1 expression in ACC may help explain these results [33,35].

In a phase 2 clinical trial, 20 patients with R/M ACC were randomized to either pembrolizumab or pembrolizumab plus RT [73]. Although the treatment was generally well tolerated, no patients had an objective response and 12/20 (60%) showed SD. In a separate phase 1b study, even among ACC patients with PD-L1-positive tumors, no objective responses were seen with pembrolizumab [74]. Nivolumab has also been trialed in this population with limited success. The phase 2 NISCAHN trial reported PR in 4/46 (9%) and SD in 26/46 (56%) patients with R/M ACC [75]. In two studies assessing the combination of nivolumab plus ipilimumab, PR was observed in only 2/32 (6%) and 1/26 (4%) of R/M ACC patients [76,77].

In summary, ICI monotherapy has produced disappointing results in R/M ACC. An ongoing clinical trial (NCT04209660) is investigating the combination of pembrolizumab plus lenvatinib in R/M salivary gland cancers including ACC, and it will be interesting to see whether there is rationale for the use of ICI in combination with other, more effective agents.

### 4.4. Biological Therapy (Non-TKI)

Vorinostat is a histone deacetylase inhibitor that has been FDA-approved for treatment-refractory cutaneous T-cell lymphoma [78]. By modulating histone and non-histone proteins, vorinostat induces apoptosis in addition to inhibiting proliferation and angiogenesis in preclinical models. In a phase 1 study in various advanced solid tumors, 1/5 patients with progressive R/M ACC showed PR and 4/5 patients had SD [79]. These very early, yet promising results then led to a phase 2 clinical trial that included only patients with R/M ACC, the majority with progressive disease prior to enrollment [80]. Among 30 patients included, the overall rate of clinical benefit was 97% (29/30), though only 2/30 experienced PR while 27/30 experienced SD. When combined with pembrolizumab in a separate clinical study, no obvious additional benefit was observed [81].

Bortezomib is an inhibitor of the 26S proteasome used most commonly in multiple myeloma, and is thought to act primarily by interfering with nuclear factor kappa B (NF-κB) complex signaling [82]. Previous work has associated increased NF-κB expression with poor prognosis in ACC, including higher propensity for vascular invasion and metastasis [83]. In a study of 25 R/M ACC patients, no objective responses were seen with bortezomib monotherapy, though 71% achieved SD [84]. In 10 of these patients, doxorubicin (which has preclinical evidence of synergy with bortezomib) was added to bortezomib at the time of progression, which resulted in PR for 1 patient and SD in 6 patients.

Lenalidomide is a molecular glue protein degrader that mediates degradation of IKZF1, IKZF3 and CK1α, and is primarily used in multiple myeloma and other hematologic malignancies [85,86]. A recent phase 1 trial tested lenalidomide combined with everolimus (an mTOR inhibitor) in various advanced solid tumors [87]. Among ACC patients, 3/15 (20%) achieved PR and 10/15 (67%) had SD. Notably, a previous phase 2 study of everolimus alone in 34 R/M ACC patients reported no objective responses [88], suggesting that lenalidomide (and other molecular glues which act through ubiquitin E3 ligases) may have activity in ACC [89].

Overall, these non-TKI biologic therapies will require additional prospective studies to determine their efficacy in R/M ACC.

## 5. Emerging Therapies for ACC

Traditional therapies for advanced head and neck cancer, such as RT and chemotherapy, have a modest response at best in ACC. Recently, there has been a shift towards defining molecular aberrations in ACC in order to develop novel, targeted therapies for this disease [53,90,91]. While many of these agents have demonstrated early promise, there is not yet sufficient data to recommend them for routine clinical use in R/M ACC.

### 5.1. Notch Signaling Pathway

The Notch signaling pathway regulates multiple cellular processes including proliferation, differentiation, and apoptosis. Because activation of Notch signaling in ACC patients is associated with a worse prognosis, the inhibition of this pathway is an active area of research [29,30]. A phase 1 clinical trial utilizing crenigacestat, a small molecule inhibitor of gamma secretase, a protein required for the activation of the Notch signaling pathway, was recently published [92]. Among patients with R/M ACC, 68% of patients achieved SD, though no objective responses were noted. A phase 2 clinical trial of AL101, a different gamma secretase inhibitor, is currently underway (NCT03691207) with promising preliminary results [53,93]. CB-103, a pan-NOTCH inhibitor which works further downstream at the transcriptional level, was recently tested in multiple solid tumors in a phase 1 trial [94,95]. Among R/M ACC patients, a median PFS of 21.7 weeks was achieved, including radiologically confirmed SD >6 months in multiple ACC patients with activating *NOTCH* mutations [95].

### 5.2. Stemness Inhibitors

Cancer stem cells represent a subpopulation of tumor cells that have stemness properties, which refers to the cell’s ability to self-renew, grow, metastasize, and continue proliferating [96]. Preclinical studies have suggested that drivers of neural crest cell stemness play a role in ACC disease progression [97]. Amcasertib (BBI503) is a novel oral cancer stemness kinase inhibitor that targets multiple serine-threonine kinases to inhibit downstream cancer stemness signaling pathways including Nanog [98]. In a phase 1b/2 clinical trial, patients with R/M ACC treated with amcasertib showed either SD, PR, or CR in 86% of patients, with 79% of patients alive at one year [99]. This medication was generally well tolerated.

### 5.3. PRMT5 Inhibitors

Protein arginine methyltransferase-5 (PRMT5) is a protein that functions via the posttranslational modification of histones and transcription factors. An increased expression of PRMT5 has been associated with poorer prognosis in several cancer types [100]. All clinical studies with PRMT5 inhibitors are currently in phase 1 trials only. First published in 2020, a phase 1 clinical trial with JNJ64619178, a selective PMRT5 inhibitor, showed only 1 PR in 54 patients with advanced solid tumors, though notably, this PR was in a patient with ACC [101]. Similarly, in a preliminary study including all solid tumor types, patients were treated with GSK3326595, a selective PRMT5 inhibitor. In those with ACC, 3/14 exhibited a PR [102]. A similar phase 1 study with PRT543, an oral PRMT5 inhibitor, reported SD in 5/7 ACC patients [103].

### 5.4. Axl Pathway Inhibitors

Among the tyrosine kinase receptors, fibroblast growth factor receptor (FGFR) overexpression is thought to play a role in multiple tumor types, including ACC. A recent preclinical study of ACC tumors found that several tumors expressed FGFR1 variants that functioned through the AXL/AKT signaling pathway, known to play a role in cancer cell survival and possibly drug resistance [104,105]. This mechanism makes the Axl pathway an interesting therapeutic target. In vitro and in vivo studies have been undertaken with ADCT-601, an antibody drug conjugate targeting AXL, which has preliminarily shown significant tumor response rates in high AXL-expressing ACC mouse models [106].

## 6. Local Therapy for Oligometastatic Lung Disease

### 6.1. Burden of Lung Metastasis in ACC

Of ACC patients who develop distant metastasis (DM), approximately 70% will have lung involvement, making it the most common site of metastasis by far [107]. Strikingly, up to 95% of patients with lung metastasis have no pulmonary symptoms at the time of diagnosis [108]. The average tumor doubling time of metastatic ACC pulmonary deposits has been reported as 393 days, which is much longer than most other malignancies [109]. Since tumors generally require about 30 doublings to reach a 1 cm diameter, some have hypothesized that a sizable portion of ACC patients may harbor pulmonary micrometastases at the time of primary diagnosis [109,110].

Long-term observational studies analyzing the natural course of metastatic ACC generally report a median survival of less than 3–5 years after appearance of distant disease [108,111,112,113,114]. However, multiple studies have suggested that the prognosis of metastatic ACC depends heavily on the specific sites of DM. In two studies that stratified ACC patients by DM site, the median survival after appearance of isolated lung metastasis was reported at 47–54 months, compared to just 19–21 months in those with bone metastases (with or without concurrent pulmonary lesions) [108,115]. While patients with isolated lung disease may have favorable short-term outcomes (with an OS around 90% at 1 year), less than one-third are estimated to survive 5 years after detection of pulmonary lesions [113].

Currently, local treatments for metastatic lesions, such as surgical removal (metastasectomy) or non-palliative RT, are not routinely recommended in ACC (NCCN category 3). Previous work from our institution found that the upfront treatment of metastatic lesions with surgery or non-palliative RT conferred no significant OS or PFS advantage compared to observation alone in a cohort of 16 R/M ACC patients [116]. In a larger report of 174 ACC patients of the National Cancer Center of China database, there was again no significant survival advantage after local therapy of metastatic lesions (via resection, RT, or both) in an unselected R/M population [114]. A recent study of 42 metastatic ACC patients suggested that those with fewer initial metastases may be better candidates for local ablative treatment [117]. However, the potential benefit of local therapies in select R/M ACC populations, such as patients with isolated small-volume lung disease, remains controversial.

### 6.2. Pulmonary Metastasectomy

Pulmonary metastasectomy (PM) is not a favored option for many metastatic cancers including HNSCC. However, PM can be considered for ACC considering its indolent nature. We identified 12 retrospective studies of PM in HNC which included ACC patients among other histologies (most commonly HNSCC), but did not separate survival outcomes for ACC, and thus, could not analyzed further [118,119,120,121,122,123,124,125,126,127,128,129]. When looking at ACC-specific post-PM survival outcomes, the results are heterogenous. There are multiple single-patient case reports of PM for ACC reporting varied clinical benefit, with differences in survival largely dictated by the development of subsequent distinct lung lesions or new metastases at non-pulmonary sites [130,131,132,133]. We identified 10 studies which included multiple ACC patients undergoing PM and reported ACC-specific survival data (as compared to the 12 reports above that presented aggregate outcomes across histologies). These studies, ranging from *n* = 3 to *n* = 109, are detailed in Table 1.

Among these 10 publications, the most impressive survival results to date are from a 2020 study including 11 ACC PM patients, which demonstrated 100% OS at both 3 years and 5 years post-PM (compared to just 62.5% and 44.6% for SCC patients at 3 and 5 years, respectively) [139]. Remarkably, a 100% 5-year OS was also reported in two additional smaller studies (*n* = 5 and *n* = 3) [50,142]. The remaining publications reporting 5-year OS display considerable variability. The largest ACC-specific PM study to date, by Girelli et al., evaluated OS in 109 total ACC patients [134]. OS was 66.8% at 5 years and 40.5% at 10 years, with the multivariable Cox regression identifying incomplete resection [aHR 3.68 (95% CI, 1.58–8.59)] and a disease-free interval (DFI) greater than 36 months [aHR 0.27 (95% CI, 0.12–0.59)] to be significantly associated with OS. A study of 13 ACC patients from 1988 reported a similar 5-year OS of 63% after PM [138], while a 1999 study of 16 ACC patients reported a post-PM OS of 84% at 5 years (with no difference in OS between solitary vs. multiple lesions) [137]. The most unfavorable outcomes are from a 2008 study including 6 ACC patients, in which 5-year OS was just 33.3%, though further information on these patients and their medical histories was not detailed [141].

Of the 10 studies, two presented only median OS or PFS and not survival percentages [136,140]. One was a 2005 study of 20 ACC patients, in which the median OS for patients with complete resection was 78 months post-PM, compared to 52 months in patients with an incomplete resection [136]. The PFS was 30 months and 15 months for complete vs. incomplete resection, respectively. The authors of this study identified two main factors associated with superior outcomes post-PM: a low tumor burden facilitating complete resection (defined as <6 pulmonary lesions), and a disease-free interval (DFI) ≥36 months [136]. The second group found similar results, with a median OS of 72 months in 9 ACC patients who underwent PM (compared to 62 months in 11 ACC patients who did not have PM) [140]. Of all the studies, the lowest median OS reported was 43.5 months, arising from the same 2008 publication mentioned above with significantly lower 5-year OS rates [141].

Most recently, a 2022 study which included 18 ACC patients reported 1-, 3-, and 5-year post-PM disease-free survival (DFS) rates of 88.9%, 38.9%, and 32.4%, respectively [135]. By 8 years, the DFS dropped to 0% in this cohort, demonstrating difficulty in achieving a permanent oncologic cure through PM alone. While these percentages are substantially lower than the other studies analyzed here, it is important to note that the authors only reported DFS and not OS, and it is likely that many of these patients survived significant periods of time after the appearance of their second recurrence, given the often slowly progressive nature of ACC [135].

In summary, with the exception of one publication, the 5-year OS rates in patients undergoing PM ranged from 63 to 100%. This is at least double the 5-year OS historically reported in ACC patients with untreated pulmonary metastases (which hovers around 28–35%) [108,113]. Nonetheless, the wide range of results in these PM studies highlights the importance of a rationally and rigorously selected subset of ACC patients with oligometastatic lung lesions who will benefit from PM. Based on the available evidence, this will most likely be patients in whom: (1) complete resection is feasible, and (2) there is a favorable DFI (which appears to be greater than 36 months in ACC) [134,136]. Above all, large (and ideally randomized) studies will be critical to establish whether PM affords a meaningful clinical benefit that justifies its risks.

### 6.3. Radiation for Pulmonary Metastases

Although surgical resection has historically been the preferred treatment of limited metastases, ablative radiation therapy (RT) is a non-invasive alternative that has yielded similar rates of local control and OS in retrospective studies of nonsurgical candidates with oligometastases [143]. As discussed above, PM has been used with varying success in R/M ACC patients with isolated small-volume lung disease (either a single lesion or oligometastases limited to a single lobe). Compared to the PM literature, there is a relative dearth of work examining the efficacy of RT as a method of local control in R/M ACC.

Stereotactic body radiation therapy (SBRT), otherwise known as stereotactic ablative radiotherapy (SABR), is an image-guided technique that delivers a highly conformal radiation dose in a small number of fractions and has demonstrated impressive local control rates with a low risk of high-grade toxicities [144,145]. In 2019, the landmark SABR-COMET phase 2 RCT of 99 patients with oligometastatic disease (1–5 total lesions) from a variety of primary sites demonstrated the efficacy of this modality in selected patients, reporting a doubling of PFS and a 13-month increase in OS in those randomized to SBRT compared to the standard-of-care palliative therapy [146]. When considering epithelial tumors as a whole, SBRT appears to confer a similar rate of local control but inferior PFS compared to PM, at least in the short term [147]. While multiple case reports have described the use of palliative RT in combination with cytotoxic chemotherapy [148,149] or ICI [150] for the treatment of ACC lung metastases, a role of SBRT in treating oligometastatic ACC has yet to be defined [151].

There is currently no consensus on the ideal SBRT dose and fractionation schedule for lung metastases, and protocols up to this point have been extrapolated from SBRT schemes that are used for medically inoperable, early-stage non-small cell lung cancer (NSCLC) [152]. There is particular concern in using SBRT for centrally located tumors (i.e., within 2 cm of the proximal bronchial tree and mediastinum), given an increased risk of radiation toxicity, including effusions of pleura or pericardium, decline in pulmonary function, and bronchopulmonary hemorrhage [153]. The recently published results of the phase 1 NRG-BR001 trial, which investigated SBRT for multiple oligometastatic lesions, reported favorable safety outcomes with no dose-limiting toxicities using 45 Gy in three fractions for peripheral lung metastases and 50 Gy in five fractions for central lung metastases [154]. In SABR-COMET, which allowed 1–5 metastases, peripheral lung metastases ≤3 cm were prescribed 54 Gy in three fractions. Tumors >3 cm or abutting chest wall were prescribed 55 Gy in five fractions. Central tumors were prescribed 60 Gy in eight fractions. SABR-COMET-10 is an ongoing phase 3 RCT (NCT03721341) that aims to assess the impact of SBRT in patients with 4–10 metastases [155]. Recommended SBRT doses are 20 Gy in one fraction, 30 Gy in three fractions, or 35 Gy in five fractions, which are lower than the original SABR-COMET study to minimize the risks of toxicity when treating more lesions. ACCs are radioresistant tumors, and thus, more ablative dose and fractionation schedules, such as 54 Gy in 3 fractions or 50–60 Gy in 5–8 fractions, appear to be reasonable for a limited number of lung metastases.

We identified three retrospective studies of SBRT which included ACC patients with oligometastases, all of which also included other HNC types. The largest of these studies was a 2022 publication across nine Italian centers, comparing oncologic outcomes of SBRT vs. conventional palliative RT in 37 patients with metastatic ACC (along with 27 additional non-ACC salivary malignancies) [156]. The median total dose of radiation delivered was 29 Gy in 3 fractions for the SBRT group and 30 Gy in 10 fractions for the palliative RT group. They reported a 2-year OS of 83% across all ACC patients, with no difference in OS between the cohorts but a significant benefit in time to local failure for SBRT compared to palliative RT techniques. It is important to point out that this study included patients with DM to a variety of locations including the lung, bone, and brain, but unfortunately, did not report data for each of these organ sites specifically; it is therefore difficult to draw conclusions about SBRT benefits in patients with isolated lung lesions [156].

The second study included 13 ACC patients and suffered from similar limitations, encompassing metastases to non-lung sites in addition to aggregating outcomes from all salivary gland cancer histologies together in a single group (ACC, adenocarcinoma, and mucoepidermoid carcinoma) [157]. The median total radiation dose was 48 Gy in 3–8 fractions. It is notable that this study reported impressive survival outcomes after SBRT for metastases from primary salivary gland cancers, with a 2-year OS of 95%, compared to just 35.2% in the remaining non-salivary HNCs.

The last study, which specifically investigated the role of SBRT in HNC patients with isolated lung metastases, included 10 ACC patients out of 82 in total (employing 50 Gy in 4 fractions for peripheral lesions, and 70 Gy in 10 fractions for central lesions) [158]. Like the other two studies, all non-SCC histologies (which also encompassed thyroid cancers, adenocarcinomas, and sarcomas, among others) were grouped together for analysis, with 2-year OS rates of 54.7% in SCCs compared to 77.4% in non-SCCs.

This literature review demonstrates that the existing evidence for SBRT of oligometastatic lung disease in ACC is limited to small retrospective studies. While encouraging 2-year OS rates >75% following SBRT have been reported among heterogeneous groups of HNC histologies that have included ACC, the lack of ACC-specific data is the major limitation in assessing the efficacy of SBRT for small-volume lung disease. In addition, two out of three studies identified consisted of patients with a variety of DM sites rather than isolated lung metastases, and all three studies reported relatively short-term survival outcomes at 2 years post-SBRT, compared to the 5-year follow-up routinely reported in the aforementioned PM studies. Follow-up SBRT investigation will be important to establish an ideal target dose (which ranged from medians of 30 to 70 Gy in the studies reviewed here). Excitingly, the currently recruiting SOLAR Trial (NCT04883671) aims to assess the efficacy of SBRT in ACC patients with 1–5 metastases. Patients will be randomized to either SBRT (to all sites of disease) or standard of care, with a planned 10-year follow-up regardless of the treatment regimen. These results will provide valuable data as to whether SBRT is a viable local therapy option for oligometastatic ACC patients.

## 7. Conclusions

**Figure 3:**
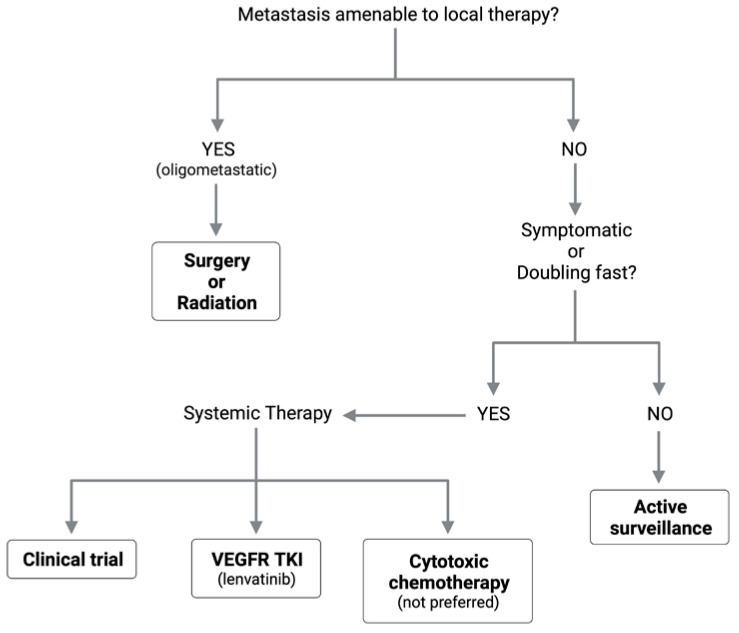
Framework for treatment modality selection based on metastatic lesion characteristics.

Despite the high burden of recurrence and metastasis in ACC, there remain no FDA-approved systemic agents and no consensus on optimal treatment regimens for these patients. In Figure 3, we present a framework for selecting a treatment modality based on the characteristics of a patient’s metastatic disease.

Among systemic therapies, studies demonstrating promising clinical benefits with VEGFR TKIs, especially lenvatinib (though also axitinib and more recently rivoceranib), have solidified the role of these agents in the R/M ACC setting. Cytotoxic chemotherapy (particularly CAP and other platinum-based combinations) has historically been employed in R/M ACC, with objective responses generally achieved in less than 20% of patients. However, the use of cytotoxic chemotherapy in ACC is based on studies with exceedingly small sample sizes, and their administration is often complicated by toxicity, so they are thus not preferred when VEGFR inhibition is feasible. To date, ICI has produced disappointing results in R/M ACC, and non-TKI biologic agents require additional investigation. Inhibition of Notch signaling, stemness, PRMT5, and Axl represent exciting emerging targets for this disease.

As pulmonary involvement occurs in 70% of patients with metastatic ACC, whether there is a role for the local treatment of lung lesions is a particularly prudent clinical question. Most retrospective studies investigating pulmonary metastasectomy (PM) in ACC patients with isolated small-volume lung disease have demonstrated favorable 5-year overall survival rates compared to historical data of patients with untreated pulmonary metastases. Predictors of the greatest PM benefit include a single metastatic focus, amenability to complete resection, and a disease-free interval ≥36 months. However, it is important to note the retrospective nature of this evidence and the lack of randomized trials assessing PM in ACC; future work with less risk of bias will be important to more definitively establish the efficacy of PM in these patients. The current evidence for stereotactic body radiation therapy (SBRT) in oligometastatic ACC is limited, as an interpretation of the few existing studies is hindered by analyses that aggregate patients with distinct HNC histologies and those with extra-pulmonary metastatic sites. Further investigation into both PM and SBRT are essential to determine whether these modalities can improve local control, survival, and quality of life in ACC patients suffering from metastatic disease.

## Figures and Tables

**Table 1:** Studies investigating pulmonary metastasectomy in ACC.

Study	*n* (ACC PMs)	Intervention	Outcomes	Ref.
Girelli et al. (2017)	109	PM: 83.5% CR 16.5% IR	5-year OS: 66.8% 10-year OS: 40.5%	[134]
Park et al. (2022)	18	PM: 100% CR	1-year DFS: 88.9% 3-year DFS: 38.9% 5-year DFS: 32.4% 8-year DFS: 0%	[135]
Locati et al. (2005)	20	PM: 55% CR 45% IR	Median OS: 78 mo. (CR) vs. 52 mo. (IR) Median PFS: 30 mo. (CR) vs. 15 mo. (IR)	[136]
Liu et al. (1999)	16	PM: 81% CR 19% IR	5-year OS: 84%	[137]
Mazer et al. (1988)	13	PM	5-year OS: 63%	[138]
AlShammari et al. (2020)	11	PM	5-year OS: 100%	[139]
Bobbio et al. (2008)	9	PM	Median OS: 72 mo.	[140]
Winter et al. (2008)	6	PM	5-year OS: 33.3% Median OS: 43.5 mo.	[141]
Ishida et al. (2020)	5	PM	5-year OS: 100%	[50]
Lu et al. (2019)	3	PM	2-year OS: 100% 5-year OS: 100%	[142]

Abbreviations: ACC, adenoid cystic carcinoma; CR, complete resection; DFI, disease-free interval; DFS, disease-free survival; HNC, head and neck cancer; IR, incomplete resection; mo., months; OS, overall survival; PFS, progression-free survival; PM, pulmonary metastasectomy.

## Abbreviations

ACC	adenoid cystic carcinoma
CAP	cyclophosphamide-doxorubicin-cisplatin
CR	complete response
DFI	disease-free interval
DFS	disease-free survival
DM	distant metastasis
HNC	head and neck cancer
HNSCC	head and neck squamous cell carcinoma
ICI	immune checkpoint inhibitor
OS	overall survival
PD-1	programmed death-1 receptor
PD-L	programmed death ligand
PFS	progression-free survival
PM	pulmonary metastasectomy
PR	partial response
R/M	recurrent/metastatic
RT	radiation therapy
SBRT	stereotactic body radiation therapy
SCC	squamous cell carcinoma
SD	stable disease
TKI	tyrosine kinase inhibitor
VEGFR	vascular endothelial growth factor receptor
